# Expression profiling of TRIM protein family in THP1-derived macrophages following TLR stimulation

**DOI:** 10.1038/srep42781

**Published:** 2017-02-17

**Authors:** Mei-Xiu Jiang, Xuan Hong, Bin-Bin Liao, Shui-Zhen Shi, Xiao-Fang Lai, Huai-Yu Zheng, Lin Xie, Yuan Wang, Xiao-Lei Wang, Hong-Bo Xin, Mingui Fu, Ke-Yu Deng

**Affiliations:** 1 Institute of Translational Medicine, Nanchang University, Nanchang, Jiangxi, 330031, China; 2 Department of Basic Medical Science, Shock/Trauma Research Center, School of Medicine, University of Missouri Kansas City, Kansas City, MO, 64108, USA

## Abstract

Activated macrophages play an important role in many inflammatory diseases including septic shock and atherosclerosis. However, the molecular mechanisms limiting macrophage activation are not completely understood. Members of the tripartite motif (TRIM) family have recently emerged as important players in innate immunity and antivirus. Here, we systematically analyzed mRNA expressions of representative TRIM molecules in human THP1-derived macrophages activated by different toll-like receptor (TLR) ligands. Twenty-nine TRIM members were highly induced (>3 fold) by one or more TLR ligands, among which 19 of them belong to TRIM C-IV subgroup. Besides TRIM21, TRIM22 and TRIM38 were shown to be upregulated by TLR3 and TLR4 ligands as previous reported, we identified a novel group of TRIM genes (TRIM14, 15, 31, 34, 43, 48, 49, 51 and 61) that were significantly up-regulated by TLR3 and TLR4 ligands. In contrast, the expression of TRIM59 was down-regulated by TLR3 and TLR4 ligands in both human and mouse macrophages. The alternations of the TRIM proteins were confirmed by Western blot. Finally, overexpression of TRIM59 significantly suppressed LPS-induced macrophage activation, whereas siRNA-mediated knockdown of TRIM59 enhanced LPS-induced macrophage activation. Taken together, the study provided an insight into the TLR ligands-induced expressions of TRIM family in macrophages.

Macrophages are the major components of innate immunity that enable the body to combat bacteria and other pathogens. However, over-activation of macrophages plays a central role in a variety of inflammatory diseases, such as septic shock, atherosclerosis, arthritis and inflammatory bowel diseases. In these disease settings, activated macrophages elaborate a large array of cytokines, growth factors and proteolytic enzymes that are critical for tissue damage and repair^1^,^2^. Macrophages are activated in response to the pathogen-associated molecular patterns by various pattern-recognition receptors (PRRs), such as the Toll-like receptors (TLRs) and the RIG-I-like receptors (RLR)^3^,^4^. There are 13 TLRs that sense various pathogen components and trigger intracellular signaling pathways that eventually mediate the induction of inflammatory cytokines, chemokines and type I interferons, which are critical for antimicrobial activity^4^,^5^. The molecular mechanisms of regulation of macrophage activation in response to TLR ligands have been largely unknown.

Tripartite motif (TRIM) proteins contain a RING finger, one or two B-box motifs and a coiled-coil motif, and are involved in many biological processes including innate immunity, viral infection, carcinogenesis and development^6^. There are over 70 members of TRIM protein family described in humans^7^. Recently, several systematic analyses suggest that many TRIM proteins are implicated in the regulation of innate immune pathways and anti-viral activities^8^,^9^,^10^,^11^. For example, Carthagena *et al*. identified 27 of the 72 human TRIM genes are sensitive to interferon (IFN) by performing a systematic analysis of TRIM gene expressions in human primary lymphocytes and monocyte-derived macrophages in response to IFNs^10^. In addition, Rajsbaum *et al*. found that the genes encoding a subset of TRIM proteins located on chromosome 7 were up-regulated by type I IFN in macrophages/DC, suggesting that they may have anti-viral functions^11^. TRIM8 negatively regulates PIAS3-mediated repression of NF-κB by inducing translocation of PIAS3 from nucleus to cytoplasm as well as its turnover^12^,^13^,^14^, whereas TRIM16 (also known as EBBP) was reported to promote IL-1β secretion. TRIM22 is involved in anti-viral pathways by activating NF-κB signaling^15^,^16^,^17^,^18^. TRIM30 induces the lysosomal degradation of TAB2 and TAB3, thereby negatively regulating NF-κB induction in the LPS-triggered TLR4 signaling pathway^19^. TRIM21 negatively regulates TLR3, −4, −7, and −9 and RLR signaling pathways by modulating the activities of IKKs and interferon regulatory factors (IRFs)^20^,^21^. TRIM27 targets all IKKs and negatively regulates the PRR pathways^21^,^22^. CARD domain ubiquitination by TRIM25 is essential for RIG-I-mediated type I interferon induction^21^,^23^. TRIM56 facilitates double-strand DNA-stimulated interferon induction by ubiquitination of STING (stimulator of interferon genes)^21^,^24^. However, the functions of most of TRIM family members remain to be characterized.

In the present study, we systematically profiled the expressions of TRIM gene family in human THP1-derived macrophages activated by different TLR ligands. The up-regulated or down-regulated TRIM genes were further confirmed by quantitative real-time polymerase chain reaction (qRT-PCR) and Western blot analysis. The function of TRIM59 in macrophage activation was further studied.

## Results

### Expression profiling of TRIM gene family in TLR ligand-activated THP1-derived macrophages.

**Figure 1:**
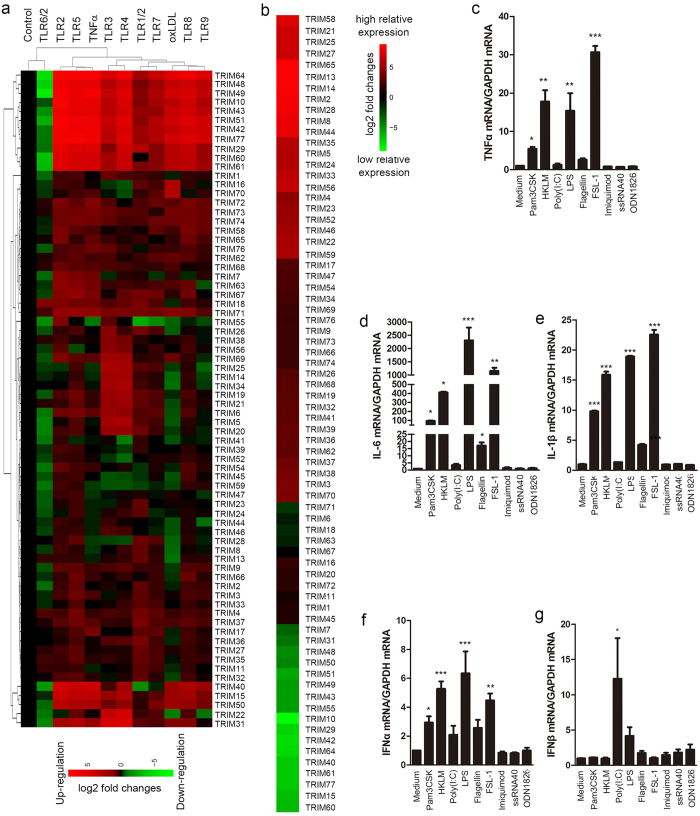
Expression profiling of TRIM gene family in TLR-triggered macrophage activation. THP1-derived macrophages were treated with the ligands for TLR1/2 (Pam3CSK, 0.2 μg/ml), TLR2 (HKLM, 2 × 10^8^ cells/ml), TLR3 (Poly(I:C), 2 μg/ml), TLR4 (LPS, 0.2 μg/ml), TLR5 (Flagellin, 0.2 μg/ml), TLR6/2 (FSL-1, 0.2 μg/ml), TLR7 (Imiquimod, 0.2 μg/ml), TLR8 (ssRNA40, 0.2 μg/ml). TLR9 (ODN1826,1 μM), TNFα (10 ng/ml) and oxLDL (20 μg/ml) for 8 hours, respectively. The total RNAs were extracted for determining the mRNA expressions of 72 TRIM genes by qRT-PCR and normalized with GAPDH mRNA (n = 2). The relative gene log2 expression changes were analyzed by hierarchical clustering using Cluster2.11 software as described in “Methods” and presented as a heat map (**a**), green: Down-regulation of gene expression; black: No change; red: Up-regulation of gene expression. (**b**) Relative expression of TRIM genes in resting THP1-derived macrophages. Mean −ΔCt values were determined by subtracting GAPDH, and each TRIM gene was normalized to the median gene expression (TRIM67), and calculated 2^−ΔΔCt^. Resulting log2 values were represented as a heat map, green: low relative expression; black: median value; Red: high relative expression. The mRNA expression of TNFα, IL-6, IL-1β (**c–e**) and IFNα, IFNβ (**f,g**) were determined by qRT-PCR and normalized with GAPDH mRNA (n = 3, *p < 0.05 **p < 0.01; ***p < 0.001 vs Control).

Macrophages are equipped with almost all TLRs, which sense different pathogens and initiate inflammatory responses. To understand the regulatory mechanisms that control macrophage activation in response to TLR ligands, we employed qRT-PCR to profile the expression changes of 72 TRIM genes in THP1-derived macrophages upon stimulation by different TLR ligands. TNFα and oxLDL were included to serve as positive controls. The primer set for 72 human TRIM gene members was tested by melting curve and product sequencing before the experiments. The entire data set were log-transformed and cluster analyzed and presented as a heat map (Fig. 1a). As shown in Fig. 1a and S1, 29 TRIM genes were highly induced (>3 fold) by one or more TLR ligands, among which 15 of them were increased by more than 10 folds compared with controls and 19 of them belong to TRIM C-IV subgroup. Moreover, the expressions of 46 TRIM members were inhibited by at least one of TLR ligands, among which 19 of them were showed an inhibition of more than 50% of control and the inhibitory ranges for other members of the TRIM family were from <75~<67% of control. Interestingly, 9 TRIM genes (TRIM10, 15, 43, 48, 49, 50, 51, 60 and 64) in TRIM C-IV subgroup, 4 genes (TRIM29, 31, 40 and 61) in TRIM C-V subgroup and TRIM42 (C-III subgroup) and TRIM77 (ungrouped) were significantly up-regulated by most TLR ligands, whereas TLR6/2 ligand almost inhibited all of the TRIM family. In addition, a group of TRIM genes which belong to C-IV subgroup of TRIM family including TRIM5, 6, 14, 20, 21, 25, 34, 38, 69 or C-V subgroup including TRIM19 and TRIM56 were markedly elevated by TLR3 and TLR4 ligands, whereas TRIM59 was down-regulated by TLR3 and TLR4 ligands in THP1-derived macrophages. TRIM21, TRIM22 and TRIM38 that were previously reported to be up-regulated during macrophage activation and negative regulators of macrophage activation were all induced by TLR3 and TLR4 ligands. In addition, TRIM8, a negative regulator of PIAS3, SOCS-1 and NF-κB signaling, was significantly down-regulated by oxLDL. Taken together, a novel group of TRIM genes (TRIM14, 15, 31, 34, 43, 48, 49, 51 and 61) were identified to be up-regulated by TLR3 and TLR4 ligands in THP1-derived macrophages. Moreover, we compared the relative expression levels of the various TRIMs in resting cells. As shown in Fig. 1b, TRIM2, 8, 13, 14, 28, 44, 58 and 65 were highly expressed in resting cells (Ct Mean <20), whereas the expressions of TRIM10, 15, 29, 40, 42, 60, 61, 64 and 77 were very low (Ct Mean >32), and the expressions of TRIM31, 43, 48, 49, 51 and 55 were relatively low (Ct Mean >30) compared with the expressions of the other members of the TRIM family.

To confirm the effectiveness of TLR ligands, we have also measured the mRNA expressions of inflammatory cytokines including TNFα, IL-6 and IL-1β. As shown in Fig. 1c–e, the mRNA expressions of TNFα, IL-6 and IL-1β were significantly induced by TLR ligands. To determine if type I interferon was involved in the regulation of TRIM genes in THP1-derived macrophages, we examined the mRNA expressions of IFNα and IFNβ with TLRs’ stimulations. As shown in Fig. 1f,g, the mRNA expressions of IFNα were induced by TLR1/2, 2, 4, 6/2 ligands, and IFNβ mRNA expressions were strongly induced by TLR3 ligand.

### Identification of one novel group of TRIM genes that were up-regulated by TLR3 and TLR4 ligands in macrophages

**Figure 2:**
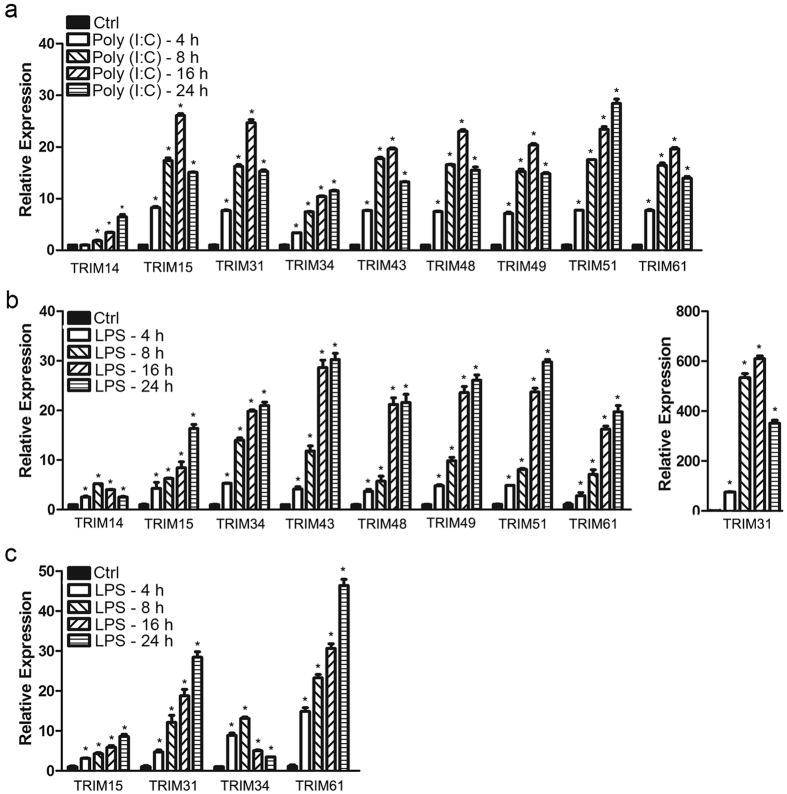
Identification of a novel group of TRIM genes that were markedly induced by TLR3 and TLR4 ligands. THP1-derived macrophages were treated with 2 μg/ml poly(I:C) (**a**) or 0.2 μg/ml LPS (**b**) in the indicated times; (**c**) Raw264.7 cells were treated with 0.2 μg/ml LPS for the indicated times. Total RNAs were extracted and the mRNA expressions of TRIM14, 15, 31, 34, 43, 48, 49, 51, 61 were determined by qRT-PCR and normalized with GAPDH mRNA. *Represents a P value < 0.01 compared with the control in the corresponding group (n = 3).

To further define the novel group of TRIM genes (TRIM14, 15, 31, 34, 43, 48, 49, 51 and 61) which were up-regulated by TLR3 and TLR4 ligands in THP1-derived macrophages, we conducted a time-course study in which the macrophages were treated with TLR3 ligand and LPS in different times. As shown in Fig. 2a, TRIM14, 15, 31, 34, 43, 48, 49, 51 and 61 mRNA were highly induced by TLR3 stimulation in THP1-derived macrophages. Their expressions were raised after 4 h of treatment, peaked at 16 h (TRIM15, 31, 43, 48, 49, 61) or 24 h (TRIM14, 34 and TRIM51), and were sustained at a high level for at least 24 h. LPS also stimulated the expressions of TRIM14, 15, 31, 34, 43, 48, 49, 51 and 61 mRNA, which were also raised after 4 h of treatment, peaked at 24 h except TRIM14 at 8 h and TRIM31 at 16 h (Fig. 2b). As shown in Fig. 2c, LPS also stimulated the expressions of TRIM15, 31, 34 and 61 mRNA in Raw264.7 cells (a murine macrophage cell line) over the time course of 4 to 24 h with a pattern similar to that in THP1-derived macrophages.

**Figure 3:**
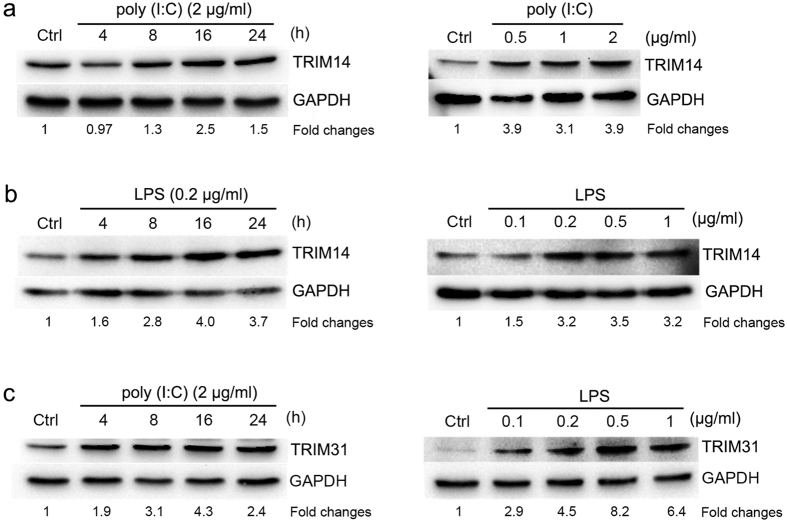
TRIM14 and TRIM31 proteins were induced by TLR3 and TLR4 ligands in dose- and time-dependent manners. The cell lysates were extracted from THP1-derived macrophages and the TRIM14 protein (**a,b**) levels were determined by Western blot with stimulations of 2 μg/ml Poly(I:C) or 0.2 μg/ml LPS at the indicated times (**a**, left; **b**, left) or at the indicated concentrations for 16 hours (**a**, right; **b**, right), and the TRIM31 proteins (**c**) were examined with stimulation of 2 μg/ml Poly(I:C) in the indicated times (**c**, left) or with LPS stimulation at the indicated concentrations for 16 h (**c**, right). The fold changes of the protein expressions were showed at the bottom of each image and GAPDH was used as an internal control.

Next, we further confirmed TLR3 and TLR4-induced expressions of TRIM14 and TRIM31 proteins by Western blot with anti-TRIM14 and anti-TRIM31 antibodies. THP1-derived macrophages were stimulated by poly (I:C) (TLR3 ligand) or LPS (TLR4 ligand) with different doses or different times. As shown in Fig. 3a, TRIM14 protein was induced by poly (I:C) in THP1-derived macrophages in dose- and time-dependent manners. Similarly, LPS also increased TRIM14 protein level in dose- and time-dependent manners in THP1-derived macrophages (Fig. 3b). In addition, our results also showed that TRIM31 protein level was increased by both TLR3 and TLR4 ligands stimulation (Fig. 3c).

### TRIM59 was down-regulated by TLR3 and TLR4 ligands in macrophages

**Figure 4:**
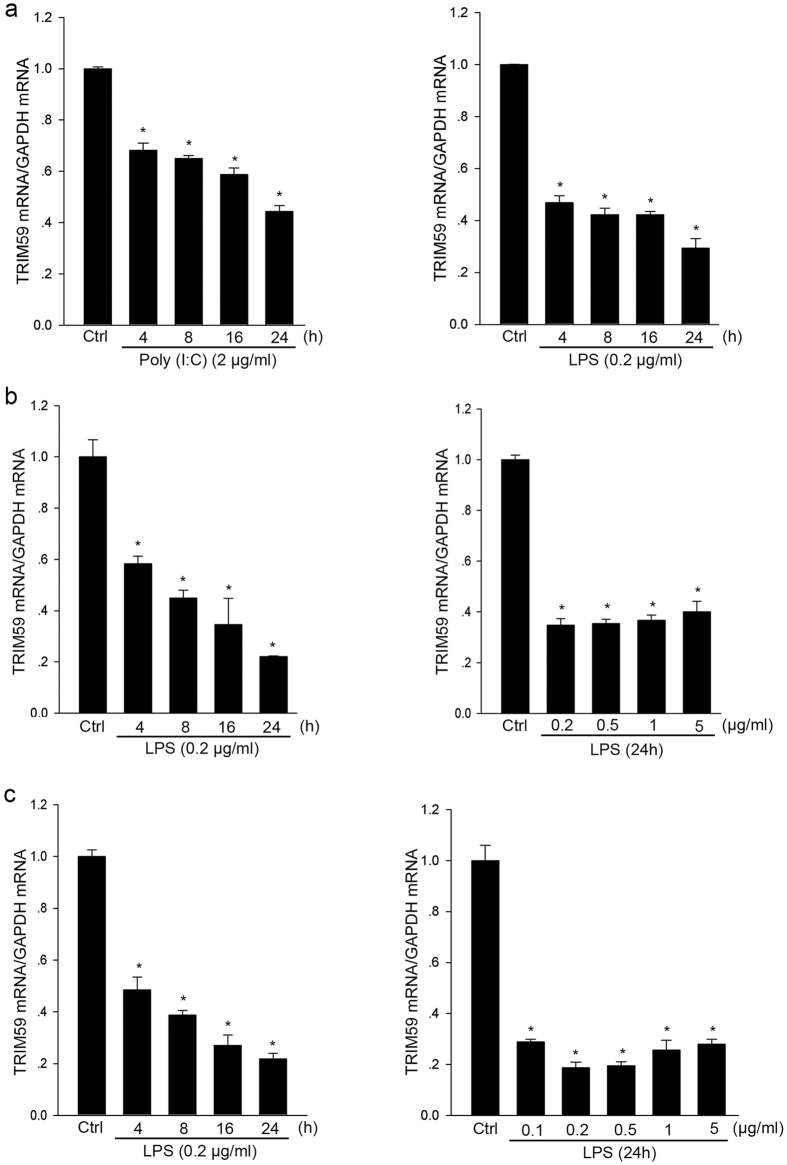
TRIM59 mRNA was down-regulated by TLR3 and TLR4 ligands in macrophages. The total RNAs were extracted from various cells and the mRNA expressions of TRIM59 were determined by qRT-PCR and normalized with GAPDH mRNA. (**a**) THP1-derived macrophages were stimulated with 2 μg/ml Poly(I:C) (**a**, left) and 0.2 μg/ml LPS (**a**, right) in the indicated times; (**b**) Raw264.7 cells were treated with 0.2 μg/ml LPS in the indicated times (**b**, left), or with LPS at the indicated concentrations for 24 h (**b**, right); (**c**) Peritoneal macrophages isolated from male C57BL/6 mice were treated with 0.2 μg/ml LPS in the indicated times (**c**, left), or with LPS at the indicated concentrations for 24 h (**c**, right). *Represents a P value < 0.05 compared with the control in the corresponding group (n = 3).

Next, we examined the expression changes of TRIM59 in THP1-derived macrophages, Raw264.7 macrophages and mouse peritoneal macrophages by qRT-PCR. As shown in Fig. 4a, TRIM59 mRNA was down-regulated by both TLR3 ligand (poly I:C) and TLR4 ligand (LPS) in THP1-derived macrophages in a time-dependent manner. Moreover, we further observed that TRIM59 mRNA was decreased by LPS in Raw264.7 cells in time-dependent and dose-dependent manners (Fig. 4b). Finally, we examined the expression changes of TRIM59 mRNA in mouse peritoneal macrophages. As shown in Fig. 4c, TRIM59 mRNA was decreased by LPS in mouse peritoneal macrophages in time-dependent and dose-dependent manners.

**Figure 5:**
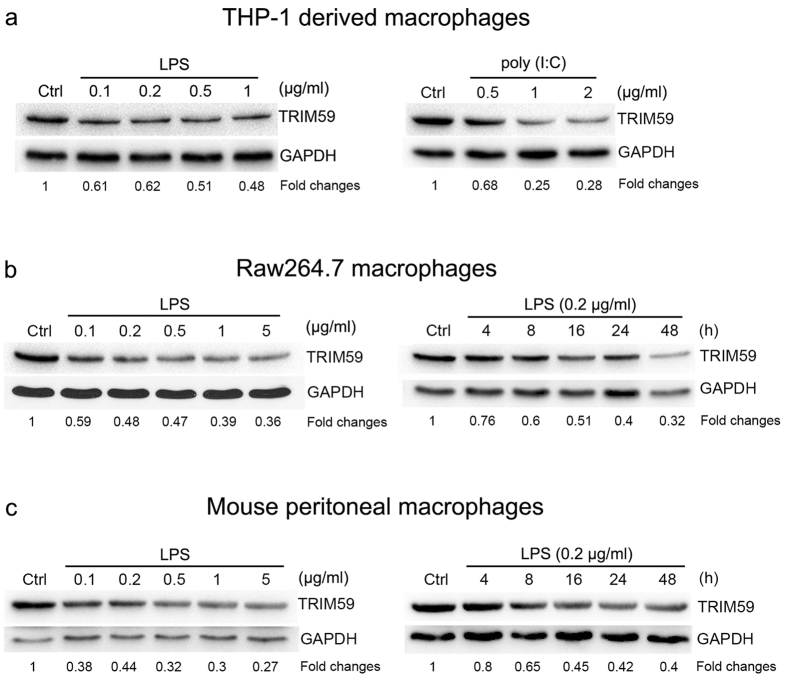
TRIM59 protein was down-regulated by TLR3 and TLR4 ligands in macrophages. The cell lysates were extracted from various cells and the expressions of TRIM59 proteins were determined by Western blot analysis and normalized with GAPDH protein. (**a**) THP1-derived macrophages were treated with 2 μg/ml Poly(I:C) (**a**, left) and 0.2 μg/ml LPS (**a**, right) in the indicated times; (**b**) Raw264.7 cells were treated with 0.2 μg/ml LPS in the indicated times (**b**, left), or with LPS at the indicated concentrations for 24 h (**b**, right); (**c**) Peritoneal macrophages isolated from male C57BL/6 mice were treated with 0.2 μg/ml LPS in the indicated times (**c**, left), or with LPS at the indicated concentrations for 24 h (**c**, right). The fold changes of the protein expression were showed at the bottom of each image and the GAPDH was used as an internal control.

To further confirm the expression changes of TRIM59 protein in human and mouse macrophages, we performed Western blot with TRIM59 antibody. As shown in Fig. 5a, TRIM59 protein level was decreased by both TLR3 ligand (poly I:C) and TLR4 ligand (LPS) in THP1-derived macrophages in a dose-dependent manner. Moreover, we further observed that TRIM59 protein was decreased by LPS in Raw264.7 cells in time-dependent and dose-dependent manners (Fig. 5b). Finally, we examined the expression changes of TRIM59 protein in mouse peritoneal macrophages. As shown in Fig. 5c, TRIM59 protein was decreased by LPS in mouse peritoneal macrophages in time-dependent and dose-dependent manners. Taken together, these results demonstrated that TRIM59 expression was down-regulated during macrophage activation.

### TRIM59 suppressed LPS-induced macrophage activation

**Figure 6:**
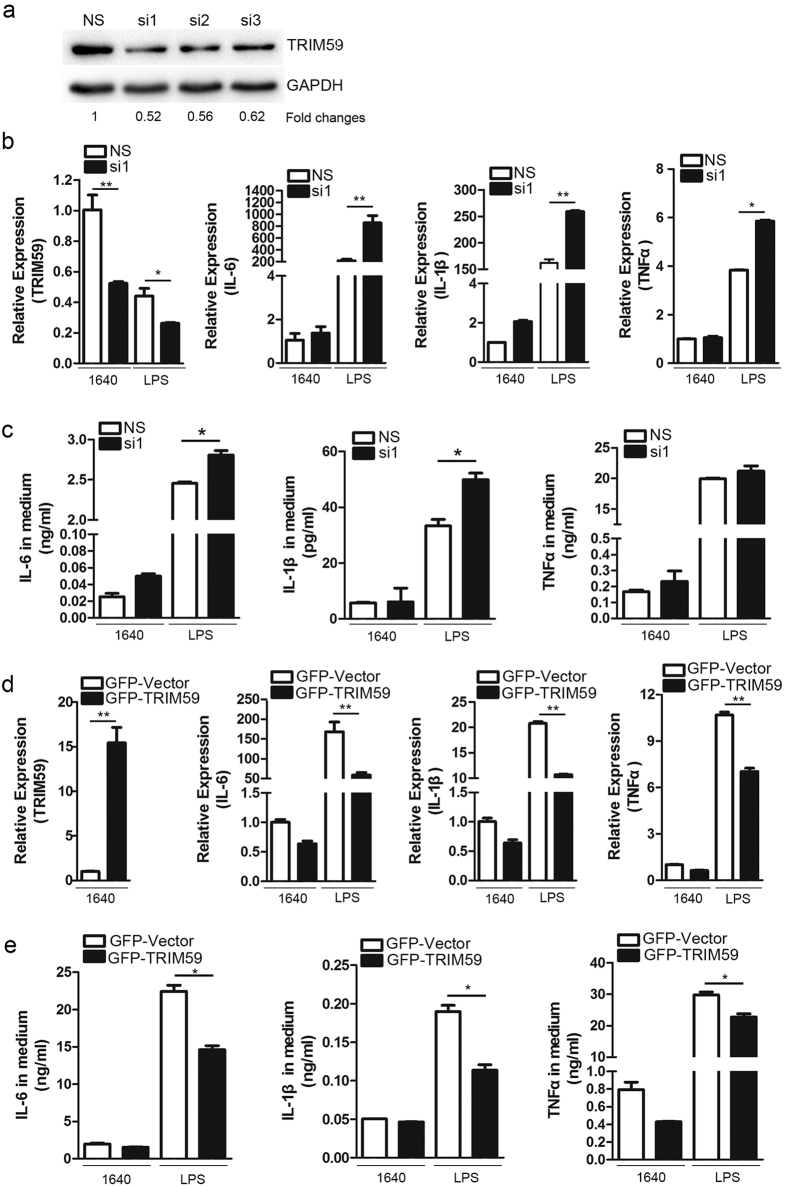
LPS-induced expressions of proinflammatory cytokines were suppressed by TRIM59 overexpression or enhanced by TRIM59 knockdown in macrophages. (**a**) Raw264.7 macrophages were transfected with 25 nM scrambled siRNA (NS) or TRIM59 siRNA1, 2, 3 for 2 days and the expressions of TRIM59 protein were determined by Western blot analysis and GAPDH was used as a control. Each experiment was repeated at least three times. (**b**) The cells were transfected with 25 nM scrambled siRNA (NS) or TRIM59 siRNA1 for 40 hours and then treated with 0.2 μg/ml LPS for 8 hours before mRNA extraction. The mRNA expressions of TRIM59 and proinflammatory cytokines including IL-6, IL-1β and TNFα were determined by qRT-PCR and normalized with GAPDH mRNA (n = 3, *p < 0.05; **p < 0.01). (**c**) The cells were transfected with 25 nM scrambled siRNA (NS) or TRIM59 siRNA1 for 24 hours and the cells were treated with 0.2 μg/ml LPS in serum-free medium for 24 hours. The concentrations of proinflammatory cytokines (IL-6, IL-1β and TNFα) in the medium were measured by ELISA (n = 3, *p < 0.05). (**d**) Peritoneal macrophages isolated from male C57BL/6 mice were transfected with 1.2 μg expression plasmid of GFP-TRIM59 and its control vector pEGFP-C2. After 40 hours, the cells were treated with 0.2 μg/ml LPS in serum-free medium for 8 hours. Total cellular RNAs were extracted and the mRNA expressions of TRIM59 and proinflammatory cytokines including IL-6, IL-1β and TNFα were determined by qRT-PCR and normalized with GAPDH mRNA (n = 3, **p < 0.01). (**e**) The peritoneal macrophages were transfected with 1.2 μg expression plasmid of GFP-TRIM59 and its control vector pEGFP-C2 for 24 hours and then the cells were treated with 0.2 μg/ml LPS for 24 hours, and the cytokine concentrations in the medium were measured by ELISA (n = 3, *p < 0.05).

Next, we examined the role of TRIM59 in macrophage activation using loss-of-function and gain-of-function strategies. Raw264.7 macrophages were transfected with 3 sets of TRIM59 siRNA at 25 nM for 2 days and then TRIM59 protein expression was determined. The results showed that TRIM59 siRNA1 (TRIM59-si1) markedly reduced TRIM59 protein expression compared with scrambled siRNA group (NS) (Fig. 6a). Therefore, TRIM59-si1 was used in the following experiments. We then examined the effect of TRIM59 knockdown on LPS-induced production of inflammatory cytokines (TNF,IL-6 and IL-1β) by qRT-PCR and ELISA. As shown in Fig. 6b,c, LPS significantly induced the expressions and secretions of IL-1β, IL-6 and TNFα, while siRNA-induced knockdown of TRIM59 markedly increased LPS-induced expressions and secretions of these inflammatory cytokines, suggesting that TRIM59 is a negative regulator of macrophage activation. To further confirm these results, the peritoneal macrophages isolated from male C57BL/6 mice were transiently transfected with expression plasmids of GFP-TRIM59 or the control vector pEGFP-C2. As shown in Fig. 6d,e, overexpression of TRIM59 significantly suppressed LPS-induced expression and secretion of IL-1β, IL-6 and TNFα. Taken together, these results indicated that TRIM59 is a negative regulator of macrophage activation.

## Discussion

TRIM protein family is a diverse protein superfamily containing a RING finger, one or two B-box motifs and a coiled-coil motif with numerous functions. In human, the TRIM family members were divided into 11 subgroups based on their C-terminal domain composition^7^,^10^,^25^. The most common C-terminal domain of TRIM proteins consists of so-called PRY-SPRY motif and is often referred as the B30.2 domain. This domain has been implicated in the ability of TRIMs to restrict the replication of certain viruses^7^,^26^. In addition to their role in innate immunity, TRIM proteins are also involved in a broad range of biological processes, including genetic disorders, neurological disorders and cancers^27^, but their function in macrophage activation are not completely understood. In this study, we employed qRT-PCR to profile the expression changes of 72 of TRIM gene family in THP1-derived macrophages activated by TLR ligands, TNFα and oxLDL. We observed that 16 TRIM genes were significantly up-regulated by 8 of 9 TLR ligands that we tested as well as TNFα and oxLDL, which include TRIM64, 48, 49, 10, 43, 51, 42, 77, 29, 60, 61, 40, 15, 50, 22 and 31. Another group of TRIM genes including TRIM38, 56, 69, 25, 14, 34, 19, 21, 6, 5, and 20 were selectively increased by TLR3 and TLR4 ligands. In contrast, TRIM59 was significantly down-regulated by TLR3 and TLR4 ligands. Interestingly, the most up-regulated TRIM genes (TRIM5, 6, 10, 14, 15, 20, 21, 22, 25, 34, 38, 43, 48, 49, 50, 51, 60, 64, 69) belong to C-IV subgroup which have the PRY-SPRY motif and a few of them are classified into C-V subgroup (TRIM19, 29, 31, 40, 56, 61), or C-III subgroup (TRIM42) and ungrouped (TRIM76, 77). The physiological significance of the expression changes of TRIM genes induced by TLR ligands warrants to be further investigated.

Carthagena and colleagues have observed that 27 of the 72 human TRIM genes are sensitive to IFN by performing a systematic analysis of TRIM gene expression in human primary lymphocytes and monocyte-derived macrophages in response to interferons^10^. They found that in MDM, type I IFN up-regulated the expressions of 16 TRIM genes (TRIM5, 6, 14, 19/PML, 20/MEFV, 21, 22, 25, 26, 31, 34, 35, 38, 56, 58 and 69) and down-regulated the expressions of 5 TRIM genes (TRIM28, 37, 54, 59 and 66). In our study, we observed that 29 TRIM members were highly induced (>3 fold) by at least one TLR ligand, whereas 46 TRIM members were inhibited (the inhibitory range from less than 50~75% of control) by at least one TLR ligand. Twelve of the up-regulated TRIM genes and 3 of the down-regulated TRIM genes in our study are consistent with Carthagena’s report. To further confirm if type I interferon was involved in the regulation of TRIM genes in macrophages, we determined the expressions of IFNα and IFNβ mRNA with the TLRs’ stimuli. We found that IFNα mRNA expressions were induced by TLR1/2, 2, 4, 6/2 ligands, and IFNβ mRNA expressions were strongly induced by TLR3 ligand in THP1-derived macrophages. These results suggest that at least some of TLR-induced TRIM genes may be mediated by the production of IFNs. However, more work would be warranted to determine this relationship.

It was reported that TRIM14 and TRIM31 were induced in human dermal fibroblast in response to retrovirus infection^28^. In this study, we found that TRIM14 and TRIM31 were up-regulated during macrophage activation. Enhanced expression of TRIM14 suppressed Sindbis virus reproduction and modulated the transcription of a large number of genes associated with innate immunity^29^. TRIM14 localizes to the outer membrane of mitochondria and interacts with MAVS. Upon viral infection, TRIM14 undergoes Lys-63-linked polyubiquitination at lys-365 and recruits NF-κB essential modulator to the MAVS signalosome, leading to the activation of both IRF3 and NF-κB pathways^30^. The role and mechanism of TRIM14 in macrophage activation need to be further investigated. Previous studies showed that TRIM31 is up-regulated in stomach cancer^31^. TRIM31 negatively regulates growth of certain cell types despite its overexpression in gastric cancer tissues^31^. Interestingly, TRIM31 was reported to interact with p52 (shc) and inhibit Src-induced anchorage-independent growth^32^. Li *et al*. recently also reported that TRIM31 is down-regulated in non-small cell lung cancer and serves as a potential tumor suppressor^33^. The role of TRIM31 in macrophage activation is not reported and need to be further investigated. TRIM15 was previously reported to play a role in the regulation of RIG-1 ligand-induced interferon production and limited stomatitis virus replication^21^. In addition, a recent report demonstrated that TRIM15 is a component of focal adhesion protein complex and controls cell migration^34^. It would be extremely interesting to find out if TRIM15 regulate macrophage migration. Currently, there are no any reports on the function of TRIM34, 43, 48, 49, 51 and 61.

TRIM59 was previously reported to act as a proto-oncogene that affects both Ras and RB signal pathways in prostate cancer models^35^. We previous reported that TRIM59 protein was significantly increased in various non-small cell lung cancer cells^36^. SiRNA-induced knockdown of TRIM59 significantly inhibited the proliferation and migration of lung cancer cells by increasing the expression of a number of cell cycle proteins including CDC25C and CDK1^36^. Here we observed that TRIM59 was significantly down-regulated during macrophage activation. In addition, overexpression of TRIM59 significantly suppressed LPS-induced expression of pro-inflammatory cytokines, whereas knockdown of TRIM59 markedly enhanced LPS-induced expression of pro-inflammatory cytokines, suggesting that TRIM59 is a novel negative regulator of macrophage activation. The mechanisms by which TRIM59 negatively regulates macrophage activation are not clear. A report suggests that TRIM59 interacts with ECSIT and negatively regulates NF-kB and IRF-3/7-mediated signal pathways^37^. However, the direct targets of TRIM59 in these signal pathways are not yet defined. Future investigations are required to determine the mechanisms by which TRIM59 interferes with the TLR3 and TLR4-induced signaling pathways.

In summary, we have identified a novel group of TRIM proteins involving in the regulation of macrophage activation. Especially, we defined TRIM59 as a negative regulator of macrophage activation. The current study not only helps to identify a functional group of TRIM proteins that may play important roles in innate immunity, but also provides the framework for future studies to dissect the functions of this emerging family.

## Methods

### Materials and animals

Various human TLR ligands were purchased from InvivoGen (USA). LPS, TNFα, phorbol 12-myristate 13-acetate (PMA), TRIM59 and TRIM31 polyclonal antibodies were obtained from Sigma (USA). TRIM14 antibody was purchased from Proteintech Group. GAPDH antibody was purchased from Santa Cruz Biotechnology (USA). All animals were treated in accordance with the Guide for the Care and Use of Laboratory Animals of Nanchang University, and all the experimental protocols were approved by the Ethics Committee of Nanchang University and the experiments were carried out in accordance with the approved guidelines.

### Cell culture

THP1 cells, a human monocyte cell line, were cultured in complete RPMI medium containing 10% fetal bovine serum (Gibco, NY, USA), 50 μg/ml of penicillin/streptomycin and 2 mM of glutamine. The cells containing 2.5~3 × 10^5^ cells/cm^2^ in 6-well plates were treated with 200 nM of phorbol 12-myristate 13-acetate (PMA) to induce the differentiation of THP1 monocytes into macrophages. After 16 h of treatment, PMA was removed and the cells were washed twice with PBS followed by incubation in complete medium for 2 days before the treatment in serum-free medium. Raw 264.7 cells, a murine macrophage cell line, were from Jihong Han lab (Nankai University, China) and cultured in complete RPMI medium. The cells were switched to serum-free medium at ~90% confluence for 2 h followed by treatment of various TLR ligands. To collect peritoneal macrophages, C57BL/6 wild type male mice with 8-week old of age were intraperitoneally (i.p.) injected with 3 ml of a 4% thioglycolate solution and maintained with access to water and normal chow for 5 days. Peritoneal macrophages were collected from the mouse abdomen by lavage with PBS. The cells were cultured in complete RPMI medium for 3 h, and then all of the floating cells were removed. The adhesive cells (macrophages) were cultured in complete RPMI medium for 2 days and switched to the serum-free medium for 2 hours, followed by treatment with various TLR ligands.

### RNA isolation, quantitative real-time PCR and clustering analysis

RNA isolation was performed as described previously^38^. RNA was quantified using a NanoDrop 2000 (Nano-drop Technologies, Wilmington, DE, USA). The cDNA was synthesized with 2 μg of total RNA using an RT kit purchased from Thermo Scientific (USA). Real time PCR was performed using an SYBR green PCR master mix purchased from Roche (USA) and PCR-specific amplification was conducted in the Applied Biosystems ViiA^TM^ real-time PCR machine (ABI, CA, USA). All primers for the 72 TRIM genes were validated using universal cDNA standards (BD Clontech). The primer sequences for all TRIM genes were summarized in Supplementary Tables 1 and 2. Quantification was performed by the deltaCT method and GAPDH was used for normalization. Normalized mRNA levels were expressed as arbitrary units by transformed the cycle times using 2^−ΔΔCt^ (see Supplementary Tables 3 and 4). Hierarchical clustering analysis was performed on the normalized, log-transformed and median-centered RNA levels by calculating Pearson correlation as distance followed by average linkage analysis using Cluster2.11 software. The resulting cluster analysis was then displayed as a tree using TreeView1.60 software.

### Protein isolation and Western blot analysis

After treatment with TLR ligands, the cells were washed twice with PBS and lysed in ice-cold lysis buffer (50 mM Tris, pH 7.5, 150 mM NaCl, 1% TritonX-100, 1% sodium deoxycholate, 1 mM PMSF, 50 mM sodium fluoride, 1 mM sodium orthovanadate, 50 μg/ml aprotinin/leupeptin). After extraction, the cellular protein levels of TRIM14, TRIM31 and TRIM59 were determined by Western blot analysis as described previously^39^ and GAPDH was used as an internal control. The expression levels of these proteins were evaluated by Quantity One software (Bio-red, USA).

### Construction of vectors and cell transfection

The TRIM59 expression vector was prepared as follows: A cDNA encoding mouse TRIM59 was generated by reverse transcription with total RNA isolated from the peritoneal macrophages and oligo(dT)18 primer. The cDNA was amplified by PCR with primers 5′-CGGGGTACCGCATGCACAATTTTGAGGAGG-3′ (forward) and 5′-TCCCCCGGGTCAACGAGAAACTATTTTCCAC-3′ (reverse). After the sequence was confirmed, the PCR product was digested with KpnI and SmaI and then subcloned into an expression vector (pEGFP-C2, Clontech, USA) to get the TRIM59 expression plasmid (GFP-TRIM59). An empty control vector (pEGFP-C2) was used as a control. For functional study, Raw264.7 cells or peritoneal macrophages isolated from male C57BL/6 mice were plated in six-well plates and transfected with scrambled siRNA/TRIM59 siRNA (purchased from RIB BI, China) or pEGFP-C2 vector/GFP-TRIM59 plasmid using HiPerFect/Effectene transfection reagent (Qiagen) in RPMI 1640 medium. After 40 hours, the cells were treated with or without LPS (0.2 μg/ml) for 8 h and then the total RNAs were isolated for determining the mRNA expressions of IL-6, IL-1β, TNFα and TRIM59. Or 24 hours later, the cells were treated with or without LPS (0.2 μg/ml) for 24 h and then the supernatants of the culture cells were collected for determining the concentrations of IL-6, IL-1β and TNFα proteins by ELISA.

### ELISA analysis

The concentrations of mouse IL-6, IL-1β and TNFα proteins in the macrophage conditioned medium were measured by ELISA analysis using the kits from MULTI SCIENCES (China) following the manufacturer’s instructions. The macrophage conditioned culture medium was diluted by 2~50 folds for the measurement. The 96-well microplates were read using a SpectraMax M5 microplate reader (Mllecular Devices, USA).

### Statistics

The data were presented as mean ± the standard error of the mean (SEM) with GraphPad Prism software 5.0. Statistical analyses were performed by one-way ANOVA followed by spss (n ≥ 3). A value of p < 0.05 was considered significant.

## Additional Information

**How to cite this article:** Jiang, M.-X. *et al*. Expression profiling of TRIM protein family in THP1-derived macrophages following TLR stimulation. *Sci. Rep.* **7**, 42781; doi: 10.1038/srep42781 (2017).

**Publisher's note:** Springer Nature remains neutral with regard to jurisdictional claims in published maps and institutional affiliations.

## Supplementary Material

Supplemental Materials

## References

[b1] Barish G. D. . A Nuclear Receptor Atlas: macrophage activation. Molecular endocrinology 19, 2466–2477, doi: 10.1210/me.2004-0529 (2005). 16051664

[b2] Burke B., Sumner S., Maitland N. & Lewis C. E. Macrophages in gene therapy: cellular delivery vehicles and *in vivo* targets. Journal of leukocyte biology 72, 417–428 (2002). 12223508

[b3] Yoneyama M. & Fujita T. Recognition of viral nucleic acids in innate immunity. Reviews in medical virology 20, 4–22, doi: 10.1002/rmv.633 (2010). 20041442

[b4] Versteeg G. A. . The E3-ligase TRIM family of proteins regulates signaling pathways triggered by innate immune pattern-recognition receptors. Immunity 38, 384–398, doi: 10.1016/j.immuni.2012.11.013 (2013). 23438823 PMC3584420

[b5] Takeuchi O. & Akira S. Pattern recognition receptors and inflammation. Cell 140, 805–820, doi: 10.1016/j.cell.2010.01.022 (2010). 20303872

[b6] Yaguchi H. . TRIM67 protein negatively regulates Ras activity through degradation of 80K-H and induces neuritogenesis. The Journal of biological chemistry 287, 12050–12059, doi: 10.1074/jbc.M111.307678 (2012). 22337885 PMC3320951

[b7] Rajsbaum R., Garcia-Sastre A. & Versteeg G. A. TRIMmunity: the roles of the TRIM E3-ubiquitin ligase family in innate antiviral immunity. Journal of molecular biology 426, 1265–1284, doi: 10.1016/j.jmb.2013.12.005 (2014). 24333484 PMC3945521

[b8] Akira S., Uematsu S. & Takeuchi O. Pathogen recognition and innate immunity. Cell 124, 783–801, doi: 10.1016/j.cell.2006.02.015 (2006). 16497588

[b9] McNab F. W., Rajsbaum R., Stoye J. P. & O’Garra A. Tripartite-motif proteins and innate immune regulation. Current opinion in immunology 23, 46–56, doi: 10.1016/j.coi.2010.10.021 (2011). 21131187

[b10] Carthagena L. . Human TRIM gene expression in response to interferons. PloS one 4, e4894, doi: 10.1371/journal.pone.0004894 (2009). 19290053 PMC2654144

[b11] Rajsbaum R., Stoye J. P. & O’Garra A. Type I interferon-dependent and -independent expression of tripartite motif proteins in immune cells. European journal of immunology 38, 619–630, doi: 10.1002/eji.200737916 (2008). 18286572

[b12] Okumura F., Matsunaga Y., Katayama Y., Nakayama K. I. & Hatakeyama S. TRIM8 modulates STAT3 activity through negative regulation of PIAS3. Journal of cell science 123, 2238–2245, doi: 10.1242/jcs.068981 (2010). 20516148

[b13] Toniato E. . TRIM8/GERP RING finger protein interacts with SOCS-1. The Journal of biological chemistry 277, 37315–37322, doi: 10.1074/jbc.M205900200 (2002). 12163497

[b14] Tomar D. . Nucleo-cytoplasmic trafficking of TRIM8, a novel oncogene, is involved in positive regulation of TNF induced NF-kappaB pathway. PloS one 7, e48662, doi: 10.1371/journal.pone.0048662 (2012). 23152791 PMC3495970

[b15] Yu S., Gao B., Duan Z., Xu W. & Xiong S. Identification of tripartite motif-containing 22 (TRIM22) as a novel NF-kappaB activator. Biochemical and biophysical research communications 410, 247–251, doi: 10.1016/j.bbrc.2011.05.124 (2011). 21651891

[b16] Barr S. D., Smiley J. R. & Bushman F. D. The interferon response inhibits HIV particle production by induction of TRIM22. PLoS pathogens 4, e1000007, doi: 10.1371/journal.ppat.1000007 (2008). 18389079 PMC2279259

[b17] Gao B., Duan Z., Xu W. & Xiong S. Tripartite motif-containing 22 inhibits the activity of hepatitis B virus core promoter, which is dependent on nuclear-located RING domain. Hepatology 50, 424–433, doi: 10.1002/hep.23011 (2009). 19585648

[b18] Hattlmann C. J., Kelly J. N. & Barr S. D. TRIM22: A Diverse and Dynamic Antiviral Protein. Molecular biology international 2012, 153415, doi: 10.1155/2012/153415 (2012). 22649727 PMC3356915

[b19] Shi M. . TRIM30 alpha negatively regulates TLR-mediated NF-kappa B activation by targeting TAB2 and TAB3 for degradation. Nature immunology 9, 369–377, doi: 10.1038/ni1577 (2008). 18345001

[b20] Oke V. & Wahren-Herlenius M. The immunobiology of Ro52 (TRIM21) in autoimmunity: a critical review. Journal of autoimmunity 39, 77–82, doi: 10.1016/j.jaut.2012.01.014 (2012). 22402340

[b21] Uchil P. D. . TRIM protein-mediated regulation of inflammatory and innate immune signaling and its association with antiretroviral activity. Journal of virology 87, 257–272, doi: 10.1128/JVI.01804-12 (2013). 23077300 PMC3536418

[b22] Zha J. . The Ret finger protein inhibits signaling mediated by the noncanonical and canonical IkappaB kinase family members. Journal of immunology 176, 1072–1080 (2006). 10.4049/jimmunol.176.2.1072 16393995

[b23] Gack M. U. . TRIM25 RING-finger E3 ubiquitin ligase is essential for RIG-I-mediated antiviral activity. Nature 446, 916–920, doi: 10.1038/nature05732 (2007). 17392790

[b24] Tsuchida T. . The ubiquitin ligase TRIM56 regulates innate immune responses to intracellular double-stranded DNA. Immunity 33, 765–776, doi: 10.1016/j.immuni.2010.10.013 (2010). 21074459

[b25] Ozato K., Shin D. M., Chang T. H. & Morse H. C. 3rd. TRIM family proteins and their emerging roles in innate immunity. Nature reviews. Immunology 8, 849–860, doi: 10.1038/nri2413 (2008). PMC3433745 18836477

[b26] Nisole S., Stoye J. P. & Saib A. TRIM family proteins: retroviral restriction and antiviral defence. Nature reviews. Microbiology 3, 799–808, doi: 10.1038/nrmicro1248 (2005). 16175175

[b27] Meroni G. & Diez-Roux G. TRIM/RBCC, a novel class of ‘single protein RING finger’ E3 ubiquitin ligases. BioEssays: news and reviews in molecular, cellular and developmental biology 27, 1147–1157, doi: 10.1002/bies.20304 (2005). 16237670

[b28] Kimsa M. W. . Differential expression of tripartite motif-containing family in normal human dermal fibroblasts in response to porcine endogenous retrovirus infection. Folia biologica 60, 144–151 (2014). 25056437 10.14712/fb2014060030144

[b29] Nenasheva V. V. . Enhanced expression of trim14 gene suppressed Sindbis virus reproduction and modulated the transcription of a large number of genes of innate immunity. Immunologic research 62, 255–262, doi: 10.1007/s12026-015-8653-1 (2015). 25948474

[b30] Zhou Z. . TRIM14 is a mitochondrial adaptor that facilitates retinoic acid-inducible gene-I-like receptor-mediated innate immune response. Proceedings of the National Academy of Sciences of the United States of America 111, E245–254, doi: 10.1073/pnas.1316941111 (2014). 24379373 PMC3896185

[b31] Sugiura T. The cellular level of TRIM31, an RBCC protein overexpressed in gastric cancer, is regulated by multiple mechanisms including the ubiquitin-proteasome system. Cell biology international 35, 657–661, doi: 10.1042/CBI20100772 (2011). 21231912

[b32] Watanabe M., Tsukiyama T. & Hatakeyama S. TRIM31 interacts with p52(Shc) and inhibits Src-induced anchorage-independent growth. Biochemical and biophysical research communications 388, 422–427, doi: 10.1016/j.bbrc.2009.08.028 (2009). 19665990

[b33] Li H. . TRIM31 is downregulated in non-small cell lung cancer and serves as a potential tumor suppressor. Tumour biology: the journal of the International Society for Oncodevelopmental Biology and Medicine 35, 5747–5752, doi: 10.1007/s13277-014-1763-x (2014). 24566900

[b34] Uchil P. D. . TRIM15 is a focal adhesion protein that regulates focal adhesion disassembly. Journal of cell science 127, 3928–3942, doi: 10.1242/jcs.143537 (2014). 25015296 PMC4163643

[b35] Valiyeva F. . Characterization of the oncogenic activity of the novel TRIM59 gene in mouse cancer models. Molecular cancer therapeutics 10, 1229–1240, doi: 10.1158/1535-7163.MCT-11-0077 (2011). 21593385

[b36] Zhan W. . TRIM59 Promotes the Proliferation and Migration of Non-Small Cell Lung Cancer Cells by Upregulating Cell Cycle Related Proteins. PloS one 10, e0142596, doi: 10.1371/journal.pone.0142596 (2015). 26599082 PMC4658198

[b37] Kondo T1., Watanabe M. & Hatakeyama S. TRIM59 interacts with ECSIT and negatively regulates NF-κB and IRF-3/7-mediated signal pathways. Biochem Biophys Res Commun. 422(3), 501–507, (Jun 8 2012). 22588174 10.1016/j.bbrc.2012.05.028

[b38] Zhang L. . DNA topoisomerase II inhibitors induce macrophage ABCA1 expression and cholesterol efflux-an LXR-dependent mechanism. Biochimica et biophysica acta 1831, 1134–1145, doi: 10.1016/j.bbalip.2013.02.007 (2013). 23466610

[b39] Jiang M. . Tamoxifen inhibits macrophage FABP4 expression through the combined effects of the GR and PPARgamma pathways. The Biochemical journal 454, 467–477, doi: 10.1042/BJ20130580 (2013). 23805908

