# Supplementary material for: Expression profiling of TRIM protein family in THP1-derived macrophages following TLR stimulation
Source: Sci Rep. 2017 Feb 17;7:42781. doi: 10.1038/srep42781 (PMC5314404; doi:10.1038/srep42781)
Supplement: Supplemental Materials [file srep42781-s1.pdf]

Expression profiling of TRIMs in THP1-derived macrophages following TLR stimulation

Mei-Xiu Jiang[1], Xuan Hong[1], Bin-Bin Liao[1], Shui-Zhen Shi[1], Xiao-Fang Lai[1], Huai-Yu Zheng[1], Lin Xie[1], Yuan Wang[1], Xiao-Lei Wang[1], Hong-Bo Xin[1,*], Mingui Fu[2,*], and Ke-Yu Deng[1,*]

Running title: TRIM gene family in THP1-derived macrophages

[1]Institute of Translational Medicine, Nanchang University, 999 Xuefu Road, Nanchang, Jiangxi 330031, China;
[2]Department of Basic Medical Science, Shock/Trauma Research Center, School of Medicine, University of Missouri Kansas City, Kansas City, MO 64108, U.S.A.

[*]**Correspondence**:
Ke-Yu Deng, MD
Institute of Translational Medicine
Nanchang University
999 Xuefu Road, Nanchang
Jiangxi 330031, China
Email: dengk26@ymail.com
Tel: +86-791-83827160

Mingui Fu, Ph.D
Department of Basic Medical Science
Shock/Trauma Research Center
School of Medicine
University of Missouri Kansas City
Kansas City, MO 64108, U.S.A.
Email: fum@umkc.edu
Tel: +1-8162355881

Hong-Bo Xin, Ph.D
Institute of Translational Medicine
Nanchang University
999 Xuefu Road, Nanchang
Jiangxi 330031, China
Email: hongboxin@yahoo.com
Tel: +86-791-83969016

Supplementary Materials

Supplementary Table 1. qRT-PCR primer sequences for human TRIM genes

Gene Name	Forward primer (5'---3')	Reverse primer (5'—3')
Trim1	CCCAGCTGGACAAAGAGAATAG	GTGGAGAACAGGTTGAGTAGAG
Trim2	CGTGTCTCTTCTCTTCGACTTC	GTCCTCCAAAGGGCTACTTATG
Trim3	CCCACGGTTGCACTTTATTTAT	GTGTAAGAAGGGTAGGGTGAAG
Trim4	CAGTGTGCCAGATACCATTGA	GAGAAGACGAGTTTGGGATGAG
Trim5	GCTCTCCGAAACCACAGATAA	CCCAGGATGCCAGTACAATAA
Trim6	CACTGGGACCTACATTCTCTTTC	CTGTAACCCAATCACCCAGTATC
Trim7	CTGAGGGTCTTGAAGAAGGAAC	GAGTTCTCAGGCCTCTGTAATG
Trim8	AATCGTGGACTTCCTGTTCTC	GACTGCGCCATCATGTTTATTAG
Trim9	CTGCCCTTCGGAGCTTATATT	CTTTGACCCTTGCCCATTTC
Trim10	GAGATCAGAAGCCAGGGATTAG	GCACTTGTGTGTGTCTGTATTT
Trim11	TGGCTGCTCTGGAAGAATTAG	TCTACTCTCTGTTCCTGTCCTC
Trim13	CCAGTCAGTGGGAAGACATAAA	AGGCCAAGCAGAAGGATTAG
Trim14	TCTCACTATCAGAGCCACAAATC	TTCAGGGTGGAGAGAGTATAGG
Trim15	CAGAGCAGGTGTGAGATGAAG	TCTCTGGGAGGGTGAGTATTT
Trim16	TAGACTCCAGGAGCCATATCC	CCAGCTACCATCAGCAGTTATT
Trim17	CGGACAGATTGAAGTGCTAAGA	CTCTCATACAGGAGGAGGTAGG
Trim18	CTGGGTGGTGAGACACAATAG	GGCGATAGAGCCGTTATCATAG
Trim19	TGGGAGCTTACTGGGTTAGA	GGATCAAGGAGGGAGGTTAATG
Trim20	CAGCCAGATCCCACATCTATAC	TGTGTTCTTCCCTCCATCAC
Trim21	CCAATCCGTGGCTGATACTT	ACCCAGGACCATAGGATAACT
Trim22	CTCGACCTGCTTATCCGTATTT	CTCAGCACAAGGGCTACTATG
Trim23	GCTCACCTTGCCTCTGTATATT	CTGTGCTTTGCTAATGTCTTTGT
Trim24	GAAGTGGCTGGACTCTCTAAAC	TGCCGTAACCGGTATGTAATC
Trim25	GGGCTTGATCTCCTGGTTATT	GTAGGAAGGGAACGCACTATTT
Trim26	CCTAAACAGGTATCCACGGAAG	CGTTGCAGAGAGAGGAGTTTAT
Trim27	GGAGGTAGAGGTGGGAGATAAA	AAAGACACTGCCCAGAATCC
Trim28	CTGCACTAGCTGTGAGGATAAT	CAGTATGGTCCTTGGTGTACTT
Trim29	GTGTCTTGACATCACCCTACC	GCAGAGGGAACAGAGGAATATC
Trim31	CTCTCGCTCTTCCTTACATTCC	GGTGGTTCCAGTCTGCTAAATA
Trim32	AGCATAGGAAGCTGGGAATAAG	GGCCAGAAGAGAGAGGTAGTA
Trim33	GTCTGTTCCGCTGACCTAAAT	GAGCACGACCTACAATCTAACC
Trim34	GTATGGTGTATTTGGCTTGAGTTATG	GCTGTGTCTTTAGGAGGGATTAG
Trim35	TGAGATAGGAGGACTGGATGAG	GGTGCCCTCTTTCCTTCTTT
Trim36	TACTCCTCCAGCTCCAGTTT	ACTCTCTACACGGTCTCTCTTC
Trim37	GAGGGAGAACTCATGGAAGATG	GGTAGCAGCGGAGCATAAA
Trim38	CTGACCCAAGAAGGGAAAGAA	CTCCCAAAGTGACAGGATTACA
Trim39	GTCAGGGAAGTGGGCTAAAG	GCAACCTTACCCTTGGAATAGA
Trim40	CCTTCATGATCCTCACCTCTTG	CACCCTATCACCTTTGCCTTAT
Trim41	CCATCTGCCTCGATTACTTCAC	ACTCATCTCTGTCCTCCTCATC

Supplementary Table 1 (Continued).

Gene Name	Forward	Reverse
Trim42	TCTGGTAGAGGAGGCATCAA	AGCACAGACAGGAGCATAAC
Trim43	GAGGATGTGAGAAGTTGGATGT	TCCCAGTAGTGTTTCCCAAAG
Trim44	TCGCTTTGTTAGGAAGGATCTG	GCCCTACCTTGCTCTCATTTAT
Trim45	GCCGTTGTCCCTAAAGATAAGA	GATGCAGACCCACACAGTATAG
Trim46	GACCCTGTCATCCTGCTTTATT	AGAGGGAGAAGAGGGACATAAG
Trim47	CTCTTCACCCACAGACTCAAG	CTCCTCTTCAGCACGGATATG
Trim48	TATGGAGAGGAGGGACTCTTTAG	CTACATGGTTGGTAGGTCTTGG
Trim49	CCTACCAGCCGAGTAGGATTAT	GGAGGTGAGAAAGAGCAATTAGG
Trim50	CCTCAGCCAAGGGTGTATTT	TGCGCAGATAGCATGGTTAG
Trim51	ACACTCACTGCAGTCTCTTTAC	TCACGGTTCTACCTTCACAATC
Trim52	CGATGAGGACGAAGATGAAGAG	GAAAGCTGCGACGTGTAAAG
Trim54	ACAGAGAGAGAGGGATAGCTAAA	CAACTTGTCTCGTCCCAGATAG
Trim55	CTCATCTGTCCCATCTGCTTAG	GTGGGCAAATACGGGTTAGA
Trim56	TCAAAGGCTGCGAAGAGAATAG	TGAGAAGCGATGGAGAATGTG
Trim58	GAGCCTGTAATCCCAGCTATTC	CTGGAGTACAGTGGTGAGATATTG
Trim59	GACACACACTGGACAGATCTTA	ACTGGAGAACAGCTTCCTTATC
Trim60	GAAGCCGTTTGGCCTTATTTC	AGACCCATTTACCAGTTCCTTATC
Trim61	ATGCAAGGAAGGATTCCCTATC	CTGCTGCTCTTCCCACTAAA
Trim62	TTCCTACTTCTCCCACTGATCT	GGTGGTGGCACATGACTATAA
Trim63	GGAAGGAAGCCAGGTTGATAC	GTCTGGGATAGGCACTGAAATAC
Trim64	TTCCTCCTTCCTCCTTCTCTT	GCTCTCGCACATAGTGTCTTAG
Trim65	TGGTGTGCGCCTGTAATC	GTAGTCCAGTGGTGCAATCTC
Trim66	GTCCATGCCCAACCTGATAA	CTGGGTGTGACCAGACATTAG
Trim67	GACCCAATGCAGGATTGATAGA	GGAGGAAGAGAGAGAGAGAACA
Trim68	GCCTCATGGAGCTGTTGTATAG	TATCATGGCACAGTGGGAAAG
Trim69	CTGGCAACCAGAGAGCTTATT	GCAGAGAGTGTCCTGCATTT
Trim70	GCACCAAATCTCCCTCATCTT	CTCTTGAAGGTTCCTGGTCATC
Trim71	GGTGAATTCTGAGGGCAAGA	CCCTCGAAGCCATACTTGTTTA
Trim72	CATGGTGAAACTCCTCTCTACTG	CCTCCTGGGTTCAAGCAATTA
Trim73	CTCCACTGGTTCCTCCATTC	GGACGTGACTTTCCTCTATCTTC
Trim74	CTCCACTGGTTCCTCCATTC	GGACGTGACTTTCCTCTATCTTC
Trim76	CAGGTACTAACCACGGAGAAAG	CCCAGTAATCGGAGGTGTATTT
Trim77	AGCCTGGACAAAGAGGAATG	GGGAGGTAGAGAAGAGACTGAA
TNFα	ATCAGAGGGCCTGTACCTCA	GGAAGACCCCTCCCAGATAG
IL-6	CCTTCCAAAGATGGCTGAAA	CAGGGGTGGTTATTGCATCT
IL-1β	ACGATGCACCTGTACGATCA	TCTTTCAACACGCAGGACAG
IFNα	TCATTGATTCGTATGCCAGCTCACC	GGATCAGTCAGCATGGTCCTCTGT
IFNβ	TTCGAAGCCTTTGCTCTGGCAC	AGATGGTCAATGCGGCGTCC
GAPDH	CCACCCATGGCAAATTCCATGGCA	TCTAGACGGCAGGTCAGGTCCACC

Supplementary Table 2. qRT-PCR primer sequences for mouse TRIM genes

Gene Name	Forward primer	Reverse primer
Trim15	AGAGATGCCTCCTGCTGAAC	TTGCTGGTTGCTGACACTTC
Trim31	CCCATCTGCATGGAAATTCT	GCTTATTGGGCCTGAACGTA
Trim34	GGCGTGAGTGGTCAGATACA	AAACTGCTTTTGGCTTCTTCA
Trim59	GAGGTCCAGCCTGTTGAAAT	CAGGAACATGGCGTTCTTTT
Trim61	ACTGGGAGGTTGATGTGGAA	TGGGAATTGTCTAGCCCAAC
GAPDH	ACCCAGAAGACTGTGGATGG	ACACATTGGGGGTAGGAACA

Supplementary Table 3. The mean cycles of qRT-PCR for TRIM genes.

**Target Name**	**Ct Mean**											
	Ctrl	TLR1	TLR2	TLR4	TLR4	TLR5	TLR6	TLR7	TLR8	TLR9	TNF	oxLDL
TRIM1	25.224	25.477	25.001	24.558	24.933	24.766	25.332	24.990	24.850	24.863	24.885	25.023
TRIM2	18.355	18.368	18.201	18.241	18.521	17.675	18.500	18.012	17.876	17.828	17.992	18.108
TRIM3	22.463	22.448	22.270	22.554	22.520	21.624	22.018	22.130	21.935	21.926	21.665	22.066
TRIM4	22.081	21.834	21.861	21.646	21.878	21.131	20.952	21.484	21.538	21.495	21.474	21.423
TRIM5	20.961	21.016	21.104	18.788	18.564	20.452	21.647	20.608	20.649	20.852	20.675	21.150
TRIM6	27.264	27.403	26.642	25.356	25.788	26.336	27.642	26.639	26.659	27.119	26.246	27.432
TRIM7	29.608	30.090	28.350	29.789	29.556	28.512	29.732	29.851	29.656	29.245	28.426	29.004
TRIM8	18.793	18.761	18.628	18.765	18.655	18.555	18.468	18.598	18.608	18.666	18.646	19.185
TRIM9	24.396	24.709	23.975	23.976	23.990	23.756	24.267	24.072	23.806	23.755	23.722	24.364
TRIM10	35.256	34.686	28.263	32.189	30.590	29.433	35.110	32.967	30.737	32.486	29.346	31.666
TRIM11	25.673	25.654	25.591	25.679	25.771	25.335	24.777	25.311	25.318	25.511	25.437	25.510
TRIM13	17.606	17.608	17.553	17.581	17.409	16.813	17.419	17.400	17.344	17.446	18.201	22.448
TRIM14	17.694	17.606	17.608	17.553	17.581	17.409	16.813	17.419	17.400	17.344	17.446	18.201
TRIM15	32.226	32.578	28.423	30.323	29.691	28.941	32.438	31.663	29.922	30.981	28.404	29.891
TRIM16	24.697	24.700	24.518	24.928	25.360	24.350	24.380	24.327	24.349	24.555	24.285	21.706
TRIM17	23.826	23.804	23.820	23.799	23.998	23.332	23.203	23.208	23.371	23.501	22.554	23.618
TRIM18	27.828	27.456	26.822	27.361	27.287	26.907	26.037	27.351	27.323	27.468	26.592	27.678
TRIM19	23.482	23.418	23.231	21.663	21.463	22.679	23.433	23.004	22.920	23.215	22.886	23.335
TRIM20	24.960	24.804	24.780	23.200	23.372	24.430	25.541	24.667	24.705	24.917	24.565	24.894
TRIM21	20.081	19.992	20.005	18.310	18.229	19.380	19.233	19.653	19.671	19.765	19.439	20.139
TRIM22	21.329	21.609	21.646	17.689	16.961	20.621	21.605	20.851	20.787	21.977	20.899	22.121
TRIM23	21.885	21.815	21.908	21.992	21.915	21.518	21.897	21.852	21.718	21.704	21.662	21.492
TRIM24	20.983	20.986	21.206	20.857	20.848	20.598	20.814	21.133	20.751	20.756	20.721	20.695
TRIM25	20.125	20.182	20.537	17.673	18.023	19.726	19.795	19.710	19.847	20.590	19.997	20.097
TRIM26	23.596	23.359	23.526	22.141	23.432	23.123	23.454	23.170	23.435	23.463	23.205	23.856
TRIM27	20.133	19.990	19.956	20.018	20.153	19.493	19.290	19.674	19.732	19.799	19.734	19.692
TRIM28	18.415	18.294	18.299	18.802	18.977	17.762	17.778	17.889	18.113	18.347	18.380	18.768
TRIM29	33.145	30.983	27.367	31.921	29.103	28.209	33.378	31.845	29.462	30.944	27.678	29.738
TRIM31	30.082	30.113	27.936	25.440	25.258	27.267	30.821	29.602	28.623	29.856	27.830	28.463
TRIM32	23.480	23.431	23.144	23.511	23.499	22.904	22.762	23.314	22.992	23.213	22.920	23.492
TRIM33	20.603	20.689	20.692	20.510	20.459	20.093	20.148	20.189	20.210	20.308	20.256	20.236
TRIM34	24.148	24.417	24.306	21.974	21.794	23.695	23.496	23.947	23.854	24.268	23.854	24.602
TRIM35	20.874	20.830	20.751	20.602	20.880	20.220	19.858	20.412	20.444	20.463	20.311	20.564
TRIM36	23.242	22.846	23.281	22.720	22.837	22.767	22.626	22.879	22.769	22.872	22.815	23.100
TRIM37	22.925	22.489	22.546	22.682	22.910	22.124	22.134	22.431	22.178	22.278	22.003	22.145
TRIM38	22.671	22.478	22.464	21.252	21.660	22.303	21.904	22.415	22.218	22.429	22.225	22.158
TRIM39	23.187	22.945	22.958	23.089	23.441	22.640	22.800	22.902	22.833	22.870	22.759	22.775
TRIM40	32.646	34.262	28.289	32.144	30.236	28.604	32.538	32.537	29.870	30.512	28.103	30.246
TRIM41	23.367	23.134	23.320	23.160	24.423	23.278	23.692	23.092	23.257	23.188	23.008	23.432

Supplementary Table 3 (Continued).

**Target Name**	**Ct Mean**											
TRIM42	33.545	31.548	26.870	30.033	28.773	27.222	33.914	30.520	28.955	29.242	26.891	29.461
TRIM43	31.208	29.519	25.105	28.003	26.642	25.371	32.213	29.197	26.848	27.767	25.284	27.019
TRIM44	18.879	18.924	18.730	18.461	18.747	18.435	18.523	18.712	18.593	19.360	18.589	18.976
TRIM45	25.229	25.481	25.074	25.610	25.566	24.593	25.129	24.902	24.664	24.870	24.833	25.321
TRIM46	21.542	21.547	21.477	20.910	21.123	20.995	21.288	21.433	21.267	21.321	21.255	21.663
TRIM47	23.946	23.862	23.812	23.857	23.809	23.318	23.933	23.745	23.766	23.878	23.817	23.415
TRIM48	30.709	29.703	24.555	27.706	26.303	24.971	34.538	28.396	26.450	27.212	24.760	26.676
TRIM49	31.121	29.820	24.613	27.370	26.108	25.050	36.558	28.229	26.193	27.309	23.999	26.474
TRIM50	30.569	30.416	27.682	29.617	28.801	27.713	30.682	29.309	28.689	29.358	27.575	28.264
TRIM51	31.544	30.140	24.652	27.719	26.174	25.006	33.650	28.403	26.253	27.162	24.973	26.952
TRIM52	21.788	21.664	21.924	21.924	22.520	21.291	21.797	21.587	21.519	21.563	21.426	21.516
TRIM54	24.006	24.177	23.759	23.908	23.836	23.399	24.627	23.814	23.520	23.729	23.427	23.974
TRIM55	31.175	33.675	30.374	30.525	31.148	30.958	32.371	32.572	31.306	31.501	31.909	31.708
TRIM56	20.492	20.348	20.341	18.999	19.388	20.717	20.454	20.139	20.120	20.362	20.179	20.338
TRIM58	19.380	19.204	18.682	19.230	19.139	18.569	19.209	18.708	18.410	18.629	18.665	18.881
TRIM59	21.222	21.523	21.280	21.763	21.827	20.707	21.779	21.269	20.982	20.940	21.127	21.452
TRIM60	32.099	31.982	27.196	29.654	28.232	27.238	35.178	30.695	28.688	30.223	27.458	28.994
TRIM61	32.627	31.584	27.642	30.345	28.936	27.997	35.481	31.329	29.340	30.285	27.585	29.498
TRIM62	23.292	23.009	22.996	22.914	23.161	22.534	22.797	22.848	22.772	22.848	22.590	22.358
TRIM63	27.548	27.257	26.222	27.293	27.217	26.131	27.397	27.250	26.834	27.830	26.298	26.807
TRIM64	33.351	31.148	25.615	28.752	27.267	25.973	38.251	29.616	27.267	28.335	25.800	27.819
TRIM65	17.868	17.586	16.992	17.559	17.783	17.456	17.431	18.007	17.092	17.254	16.854	17.356
TRIM66	24.343	24.336	24.245	24.298	24.124	23.464	24.187	24.135	23.629	23.981	23.702	24.300
TRIM67	26.387	25.505	25.177	26.329	25.709	24.559	26.353	25.704	25.580	26.170	24.948	25.946
TRIM68	23.507	23.488	22.833	23.287	23.206	22.940	22.874	23.259	23.267	23.264	22.980	22.939
TRIM69	24.447	24.518	23.312	22.534	22.402	23.849	24.473	24.215	24.063	24.541	23.872	23.551
TRIM70	22.544	22.540	22.504	22.799	23.091	22.146	22.336	21.970	22.471	22.197	22.017	19.503
TRIM71	28.174	27.416	26.554	27.393	27.218	26.444	27.317	27.329	26.832	27.547	26.504	26.774
TRIM72	24.901	24.723	23.810	24.586	24.962	24.257	24.230	24.140	23.720	24.141	23.651	24.129
TRIM73	24.393	24.107	23.608	23.838	23.920	23.536	23.821	24.216	23.393	23.561	23.371	23.340
TRIM74	24.363	24.168	23.596	23.875	24.101	23.400	23.982	23.564	23.292	23.537	23.448	23.440
TRIM76	24.461	24.625	24.180	23.846	24.212	23.615	24.743	24.148	23.998	24.182	23.598	23.889
TRIM77	32.629	30.929	25.922	29.439	27.491	25.500	33.346	29.862	27.607	28.347	26.184	28.013
GAPDH	15.680	15.845	15.840	15.713	15.729	15.564	15.029	15.693	15.703	15.554	15.511	15.422

Supplementary Table 4. The mRNA expressions of the TRIM genes were induced by TLRs ligands with qRT-PCR in human THP-1 cell derived macrophages.

	Control	TLR1/2	TLR2	TLR3	TLR4	TLR5	TLR6/2	TLR7	TLR8	TLR9	TNFa	OxLDL
TRIM1	1	1.29	1.32	1.59	1.36	1.35	0.60	1.17	1.20	1.12	0.98	0.90
TRIM2	1	1.52	1.26	1.08	0.99	1.58	0.58	1.26	1.29	1.26	1.00	0.93
TRIM3	1	1.55	1.29	0.94	1.07	1.76	0.88	1.25	1.33	1.27	1.35	1.03
TRIM4	1	1.82	1.32	1.35	1.28	1.90	1.41	1.50	1.35	1.31	1.18	1.24
TRIM5	1	1.48	1.02	4.51	5.87	1.40	0.40	1.27	1.15	0.94	0.94	0.69
TRIM6	1	1.40	1.74	3.75	3.10	1.87	0.49	1.53	1.41	0.97	1.57	0.70
TRIM7	1	1.10	2.70	0.88	1.16	2.10	0.59	0.84	0.89	1.13	1.76	1.19
TRIM8	1	1.57	1.27	1.02	1.23	1.16	0.81	1.14	1.05	0.96	0.86	0.60
TRIM9	1	1.24	1.51	1.34	1.48	1.53	0.70	1.24	1.39	1.37	1.24	0.80
TRIM10	1	2.28	144.01	8.37	28.29	55.68	0.71	4.86	21.20	5.97	46.59	9.46
TRIM11	1	1.56	1.20	1.00	1.04	1.24	1.20	1.28	1.18	0.98	0.91	0.88
TRIM13	1	1.63	1.20	1.10	1.20	1.20	1.18	1.20	1.13	1.12	0.92	0.55
TRIM14	1	1.26	0.95	4.34	5.10	1.19	0.87	1.11	1.17	0.77	0.88	0.60
TRIM15	1	1.20	15.78	3.74	6.46	9.59	0.89	1.47	4.57	2.07	10.96	3.96
TRIM16	1	1.53	1.28	0.85	0.70	1.25	0.80	1.29	1.18	0.97	1.03	6.25
TRIM17	1	1.56	1.14	1.02	0.99	1.39	0.99	1.53	1.27	1.10	1.87	0.91
TRIM18	1	1.99	2.27	1.38	1.62	1.86	2.23	1.38	1.31	1.12	1.83	0.87
TRIM19	1	1.61	1.35	3.53	4.52	1.72	0.67	1.39	1.37	1.05	1.17	0.87
TRIM20	1	1.71	1.28	3.38	3.35	1.42	0.43	1.22	1.10	0.90	1.02	0.82
TRIM21	1	1.63	1.19	3.41	4.02	1.60	1.16	1.34	1.23	1.09	1.21	0.75
TRIM22	1	1.27	0.91	12.46	23.01	1.61	0.53	1.39	1.35	0.56	1.04	0.45
TRIM23	1	1.61	1.11	0.93	1.09	1.27	0.64	1.02	1.04	0.99	0.90	1.03
TRIM24	1	1.53	0.97	1.09	1.22	1.28	0.72	0.90	1.09	1.02	0.93	0.96
TRIM25	1	1.48	0.85	5.47	4.78	1.30	0.81	1.33	1.12	0.63	0.85	0.80
TRIM26	1	1.81	1.19	2.74	1.25	1.37	0.71	1.34	1.03	0.96	1.02	0.66
TRIM27	1	1.70	1.28	1.08	1.10	1.53	1.15	1.37	1.22	1.10	1.02	1.07
TRIM28	1	1.67	1.23	0.76	0.75	1.55	1.00	1.43	1.14	0.92	0.79	0.62
TRIM29	1	6.88	62.03	2.33	18.35	30.11	0.55	2.45	11.88	4.03	34.27	8.33
TRIM31	1	1.50	5.00	24.95	31.56	6.92	0.39	1.39	2.54	1.02	3.69	2.41
TRIM32	1	1.59	1.43	0.98	1.10	1.47	1.06	1.12	1.30	1.05	1.14	0.78
TRIM33	1	1.45	1.06	1.07	1.23	1.40	0.88	1.33	1.21	1.07	0.99	1.01
TRIM34	1	1.28	1.01	4.51	5.70	1.35	1.01	1.14	1.13	0.81	0.95	0.57
TRIM35	1	1.58	1.23	1.21	1.11	1.55	1.30	1.37	1.25	1.16	1.14	0.97
TRIM36	1	2.02	1.10	1.43	1.48	1.37	0.99	1.28	1.28	1.13	1.04	0.87
TRIM37	1	2.08	1.47	1.18	1.13	1.71	1.11	1.40	1.55	1.37	1.47	1.35
TRIM38	1	1.16	1.31	2.43	1.92	1.08	1.12	1.00	1.22	0. 96	1.03	1.08
TRIM39	1	1.19	1.33	0.97	0.80	1.22	0.86	1.02	1.14	1.01	1.02	1.00
TRIM40	1	0.33	23.28	1.29	5.06	13.78	0.28	0.90	6.12	3.56	17.63	3.99

**TRIM41**	1	1.19	1.17	1.05	0.46	0.89	0.53	1.01	0.96	0.92	0.97	0.72
**TRIM42**	1	4.03	116.08	10.37	26.03	67.00	0.51	6.81	21.53	16.02	76.16	12.81
**TRIM43**	1	3.26	78.09	8.39	22.56	47.84	0.33	3.37	18.35	8.82	45.92	13.78
**TRIM44**	1	0.98	1.26	1.22	1.04	1.14	0.84	0.94	1.09	0.58	0.92	0.71
**TRIM45**	1	0.85	1.26	0.70	0.75	1.30	0.71	1.05	1.32	1.04	1.00	0.71
**TRIM46**	1	1.01	1.19	1.41	1.27	1.22	0.78	0.90	1.08	0.95	0.92	0.69
**TRIM47**	1	1.07	1.25	0.97	1.05	1.29	0.66	0.96	1.01	0.85	0.83	1.09
**TRIM48**	1	2.03	80.90	7.29	20.20	44.66	0.05	4.16	17.11	9.16	46.72	12.36
**TRIM49**	1	2.49	103.39	12.24	30.76	56.26	0.02	6.21	27.21	11.40	105.35	18.92
**TRIM50**	1	1.12	8.40	1.76	3.24	6.06	0.61	2.00	3.29	1.88	6.03	3.73
**TRIM51**	1	2.67	134.92	12.89	39.40	77.76	0.15	7.38	34.99	16.92	71.90	18.21
**TRIM52**	1	1.02	1.21	0.86	0.59	1.20	0.77	1.10	1.20	1.09	1.05	1.10
**TRIM54**	1	0.83	1.58	1.01	1.10	1.29	0.50	1.10	1.40	1.13	1.23	0.93
**TRIM55**	1	0.17	2.31	1.48	0.99	0.99	0.34	0.36	0.91	0.75	0.49	0.63
**TRIM56**	1	1.03	1.48	2.66	2.10	0.73	0.79	1.23	1.29	1.02	1.02	1.01
**TRIM58**	1	1.06	2.16	1.05	1.15	1.49	0.87	1.53	1.95	1.58	1.35	1.29
**TRIM59**	1	0.76	1.28	0.70	0.71	1.21	0.52	0.93	1.18	1.14	0.81	0.78
**TRIM60**	1	1.01	39.75	5.14	14.23	24.65	0.09	2.54	10.59	3.43	20.46	7.82
**TRIM61**	1	2.12	35.36	4.97	13.12	22.09	0.11	2.66	9.91	4.45	26.51	7.24
**TRIM62**	1	1.25	1.37	1.33	1.11	1.51	1.07	1.47	1.46	1.19	1.31	1.58
**TRIM63**	1	1.26	2.80	1.22	1.28	2.38	0.84	1.33	1.67	0.72	1.91	1.38
**TRIM64**	1	4.74	237.99	24.75	68.89	148.38	0.03	14.39	68.87	28.41	150.87	38.28
**TRIM65**	1	1.25	2.05	1.27	1.08	1.19	1.03	0.98	1.74	1.34	1.62	1.18
**TRIM66**	1	1.03	1.19	1.05	1.18	1.64	0.85	1.25	1.66	1.13	1.25	0.85
**TRIM67**	1	1.90	2.58	1.06	1.62	3.17	0.78	1.73	1.78	1.02	2.18	1.12
**TRIM68**	1	1.19	1.66	1.34	1.31	1.48	1.12	1.19	1.05	1.10	1.41	1.36
**TRIM69**	1	1.11	2.28	4.35	4.39	1.51	0.71	1.18	1.17	0.87	1.46	1.70
**TRIM70**	1	1.17	1.07	0.97	0.73	1.32	0.83	1.49	0.94	1.19	1.41	7.54
**TRIM71**	1	1.98	3.19	1.98	2.07	3.32	1.30	1.80	2.26	1.44	3.11	2.42
**TRIM72**	1	1.32	2.21	1.44	1.02	1.56	1.15	1.70	2.02	1.58	2.33	1.56
**TRIM73**	1	1.43	1.79	1.69	1.48	1.81	1.07	1.13	1.79	1.66	1.99	1.90
**TRIM74**	1	1.34	1.77	1.62	1.28	1.95	0.94	1.74	1.88	1.65	1.84	1.74
**TRIM76**	1	0.95	1.32	1.57	1.22	1.47	0.67	1.26	1.38	1.25	1.78	1.25
**TRIM77**	1	3.46	113.71	9.37	36.15	114.48	0.49	6.88	32.55	19.98	85.32	20.71

Figure S1. Summary of TRIM expressions in human macrophages upon TLR ligands, TNFa and oxLDL stimuli. TRIM members are classified into 11 subgroups as defined by Ozato et al and Ricardo Rajsbaumet al.

sub-group	proteins	TLR1/2	TLR2	TLR3	TLR4	TLR5	TLR6/2	TLR7	TLR8	TLR9	TNFa	OxLDL
I	TRIM1	0	0	+/-	0	0	--	0	0	0	0	0
VII	TRIM2	+/-	0	0	0	+/-	--	0	0	0	0	0
VII	TRIM3	+/-	0	0	0	+/-	0	0	0	0	0	0
IV	TRIM4	+/-	0	0	0	+/-	0	+/-	0	0	0	0
IV	TRIM5	0	0	+	++	0	--	+/-	0	0	0	-
IV	TRIM6	0	+/-	+	+	+/-	--	+/-	0	0	+/-	-
IV	TRIM7	0	+/-	0	0	+/-	--	0	0	0	+/-	0
V	TRIM8	+/-	0	0	0	0	0	0	0	0	0	--
I	TRIM9	0	+/-	0	0	+/-	-	0	0	0	0	0
IV	TRIM10	+/-	+++	++	+++	+++	-	+	+++	++	+++	++
IV	TRIM11	+/-	0	0	0	0	0	0	0	0	0	0
XI	TRIM13	+/-	0	0	0	0	0	0	0	0	0	--
IV	TRIM14	0	0	+	++	0	0	0	0	0	0	--
IV	TRIM15	0	+++	+	++	++	0	0	+	+/-	+++	+
IV	TRIM16	+/-	0	0	-	0	0	0	0	0	0	++
IV	TRIM17	+/-	0	0	0	0	0	+/-	0	0	+/-	0
I	TRIM18	+/-	+/-	0	+/-	+/-	+/-	0	0	0	+/-	0
V	TRIM19	+/-	0	+	+	+/-	--	0	0	0	0	0
IV	TRIM20	+/-	0	+	+	0	--	0	0	0	0	0
IV	TRIM21	+/-	0	+	+	+/-	0	0	0	0	0	0
IV	TRIM22	0	0	+++	+++	0	--	0	0	--	0	--
IX	TRIM23	+/-	0	0	0	0	--	0	0	0	0	0
VI	TRIM24	+/-	0	0	0	0	-	0	0	0	0	0
IV	TRIM25	0	0	++	+	0	0	0	0	--	0	0
IV	TRIM26	+/-	0	+/-	0	0	-	0	0	0	0	--
IV	TRIM27	+/-	0	0	0	+/-	0	0	0	0	0	0
VI	TRIM28	+/-	0	0	0	+/-	0	0	0	0	0	--
V	TRIM29	++	+++	+/-	+++	+++	--	+/-	+++	+	+++	++
V	TRIM31	+/-	++	+++	+++	++	--	0	+/-	0	+	+/-
VII	TRIM32	+/-	0	0	0	0	0	0	0	0	0	0
VI	TRIM33	0	0	0	0	0	0	0	0	0	0	0
IV	TRIM34	0	0	+	++	0	0	0	0	0	0	--
IV	TRIM35	+/-	0	0	0	+/-	0	0	0	0	0	0
I	TRIM36	+/-	0	0	0	0	0	0	0	0	0	0
VIII	TRIM37	+/-	0	0	0	+/-	0	0	+/-	0	0	0
IV	TRIM38	0	0	+/-	+/-	0	0	0	0	0	0	0

sub-group	proteins	TLR1/2	TLR2	TLR3	TLR4	TLR5	TLR6/2	TLR7	TLR8	TLR9	TNFa	OxLDL
IV	TRIM39	0	0	0	0	0	0	0	0	0	0	0
V	TRIM40	--	+++	0	++	+++	--	0	+++	+	+++	+
IV	TRIM41	0	0	0	--	0	--	0	0	0	0	-
III	TRIM42	+	+++	+++	+++	+++	--	++	+++	+++	+++	+++
IV	TRIM43	+	+++	++	+++	+++	--	+	+++	++	+++	+++
V	TRIM44	0	0	0	0	0	0	0	0	--	0	-
X	TRIM45	0	0	-	0	0	-	0	0	0	0	-
I	TRIM46	0	0	0	0	0	0	0	0	0	0	-
IV	TRIM47	0	0	0	0	0	--	0	0	0	0	0
IV	TRIM48	+/-	+++	++	+++	+++	--	+	+++	++	+++	+++
IV	TRIM49	+/-	+++	+++	+++	+++	--	++	+++	+++	+++	+++
IV	TRIM50	0	++	+/-	+	++	--	+/-	+	+/-	++	+
IV	TRIM51	+/-	+++	+++	+++	+++	--	++	+++	+++	+++	+++
V	TRIM52	0	0	0	--	0	0	0	0	0	0	0
II	TRIM54	0	+/-	0	0	0	--	0	0	0	0	0
II	TRIM55	--	+/-	0	0	0	--	--	0	-	--	--
V	TRIM56	0	0	+/-	+/-	-	0	0	0	0	0	0
IV	TRIM58	0	+/-	0	0	0	0	+/-	+/-	+/-	0	0
XI	TRIM59	0	0	-	-	0	--	0	0	0	0	0
IV	TRIM60	0	+++	++	+++	+++	--	+/-	+++	+	+++	++
V	TRIM61	+/-	+++	+	+++	+++	--	+/-	++	+	+++	++
IV	TRIM62	0	0	0	0	+/-	0	0	++	0	0	+/-
II	TRIM63	0	+/-	0	0	+/-	0	0	+/-	-	+/-	0
IV	TRIM64	+	+++	+++	+++	+++	--	+++	+++	+++	+++	+++
IV	TRIM65	0	+/-	0	0	0	0	0	+/-	0	+/-	0
VI	TRIM66	0	0	0	0	+/-	0	0	+/-	0	0	0
I	TRIM67	+/-	+/-	0	+/-	+	0	+/-	+/-	0	+/-	0
IV	TRIM68	0	+/-	0	0	0	0	0	0	0	0	0
IV	TRIM69	0	+/-	+	+	+/-	-	0	0	0	0	+/-
IV	TRIM70	0	0	0	-	0	0	0	0	0	0	++
VII	TRIM71	+/-	+	+/-	+/-	+	0	+/-	+/-	0	+	+/-
IV	TRIM72	0	+/-	0	0	+/-	0	+/-	+/-	+/-	+/-	+/-
V	TRIM73	0	+/-	+/-	0	+/-	0	0	+/-	+/-	+/-	+/-
V	TRIM74	0	+/-	+/-	0	+/-	0	+/-	+/-	+/-	+/-	+/-
	TRIM76	0	0	+/-	0	0	--	0	0	0	+/-	0
	TRIM77	+	+++	++	+++	+++	--	++	+++	+++	+++	+++

Keys:

TRIM expression

Symbol	Fold change	
+++	>10	Up-regulation
++	5-10	Up-regulation
+	3-5	Up-regulation
+/-	1.5-3	
0	No change	
--	<0.67	Down-regulation
-	0.67-0.75	Down-regulation

TRIM expression

I	IV	VII	X
II	V	VIII	XI
III	VI	IX	

Defferent subgroup of TRIM family
